# Non-coding RNAs in neuropathic pain

**DOI:** 10.1042/NS20190099

**Published:** 2020-04-23

**Authors:** Theodora Kalpachidou, Kai K. Kummer, Michaela Kress

**Affiliations:** Institute of Physiology, Medical University of Innsbruck, Innsbruck, Austria

**Keywords:** lncRNA, microRNA, neuroimmune interations, neuropathic pain, non-coding RNA

## Abstract

Neuro-immune alterations in the peripheral and central nervous system play a role in the pathophysiology of chronic pain in general, and members of the non-coding RNA (ncRNA) family, specifically the short, 22 nucleotide microRNAs (miRNAs) and the long non-coding RNAs (lncRNAs) act as master switches orchestrating both immune as well as neuronal processes. Several chronic disorders reveal unique ncRNA expression signatures, which recently generated big hopes for new perspectives for the development of diagnostic applications. lncRNAs may offer perspectives as candidates indicative of neuropathic pain in liquid biopsies. Numerous studies have provided novel mechanistic insight into the role of miRNAs in the molecular sequelae involved in the pathogenesis of neuropathic pain along the entire pain pathway. Specific processes within neurons, immune cells, and glia as the cellular components of the neuropathic pain triad and the communication paths between them are controlled by specific miRNAs. Therefore, nucleotide sequences mimicking or antagonizing miRNA actions can provide novel therapeutic strategies for pain treatment, provided their human homologues serve the same or similar functions. Increasing evidence also sheds light on the function of lncRNAs, which converge so far mainly on purinergic signalling pathways both in neurons and glia, and possibly even other ncRNA species that have not been explored so far.

## Introduction

Human neuropathic pain disorders are difficult to diagnose and treat due to their diversity, which even increases with the development of chronic pain. The most frequent neuropathic pain disorder is diabetic painful neuropathy (DPN), which occurs as a common complication of diabetes mellitus [1,2]. Although good control of blood glucose levels can reduce the incidence of DPN mainly in Type I diabetes, more than half of the patients still develop DPN for which only symptomatic therapy of low to moderate efficacy is available to date [3]. Neuroinflammatory signatures have been identified as critical components of DPN but its complex pathogenesis is still incompletely understood [3,4]. Pathological neuro-immune communication has, likewise, been associated with other painful neuropathies such as neuropathic pain occurring in up to 50% of patients experiencing traumatic nerve injury as a consequence of accidents, warfare or surgical procedures [5–7]. Also the neurogenic complex regional pain syndrome (CRPS), an enigmatic complication of bone fracture or tissue injury, is associated with neuro-inflammatory deficits [8]. In the majority of patients symptoms largely resolve, however in 30% of cases the pain persists or even intensifies [9]. The beneficial effect of glucocorticosteroids in acute CRPS points towards pathophysiological mechanisms associated with neuro-immune dysfunction [9–11].

Such neuro-immune alterations in the peripheral and central nervous system play a role in the pathophysiology of chronic pain in general, and members of the non-coding RNA (ncRNA) family, specifically the short, 22 nucleotide microRNAs (miRNAs) as regulators of gene expression act as master switches orchestrating both immune and neuronal processes. The long non-coding RNAs (lncRNAs) can regulate gene expression but when containing multiple miRNA-binding elements can serve as endogenous sponges neutralizing these miRNAs. Several chronic disorders reveal unique miRNA and lncRNA expression signatures, which recently generated big hopes for new perspectives for the development of diagnostic applications. ncRNAs modulating both neuronal and immune processes further promise therapeutic potential for diseases with a neuro-immune component [12,13]. Specifically, ncRNAs may regulate neuro-immune communication signals in the pain pathway by controlling macromolecular complexes in neurons, glia and immune cells. Understanding the concerted function of miRNA and lncRNAs in the regulation of nociceptive transduction and action potential generation, the synaptic transmission in the spinal dorsal horn and brain, the intercellular communication between neurons and non-neuronal cells, such as microglia, and the endogenous inhibitory control circuits, and defining their importance in the brain circuitries connected to cognitive, emotional and behavioural components involved in pain will shed new light on the so far enigmatic pathophysiology of neuropathic pain disorders. This review will focus on miRNAs and lncRNAs, as a large amount of literature suggests important and partially opposing roles for these ncRNAs in the establishment and chronification of neuropathic pain.

## microRNA

### Generation of miRNA

Pain conditions have been associated with deregulated miRNA expression from primary afferent nociceptors to brain areas associated with emotional components of pain perception [14–19]. Unique signatures of ncRNAs are associated with altered innate immune signalling and secreted miRNAs are even considered a new form of neuro-immune communication, and control immune cell activity as well as neuron function [13,20–22]. Thus, ncRNAs may act as essential modulators of processes for the establishment and maintenance of neuropathic pain.

**Figure 1:**
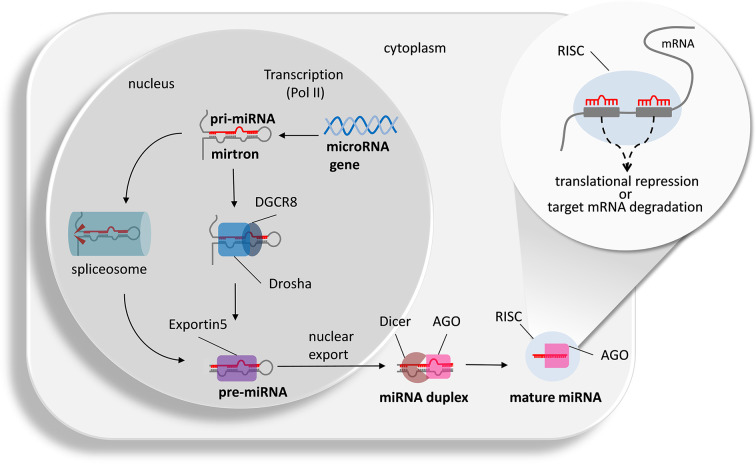
Classical and alternative mechanisms of mature miRNA generation and their action The microRNA gene is transcribed into a primary microRNA (pri-microRNA) or mirtron by polymerase II (Pol II). Drosha together with the accessory protein DGCR8 cleaves the pri-miRNA into a pre-miRNA. Alternatively, the pre-miRNA can arise from alternative splicing of the mirtron. Pre-miRNA binds to Exportin5 for nuclear export. Within the cytoplasm, Dicer and Argonaute 2 (AGO) cleave off the hairpin structure to generate a duplex miRNA. The two strands separate and within the RNA silencing complex (RISC) and in the presence of AGO the mature miRNA hybridizes with complementary seed sequences (8 nucleotides length) of possible target RNA strands (adapted from [32,33]). Depending on the degree of homology, the miRNA induces translational repression (incomplete match) or target mRNA degradation (full match, not in mammals) is induced.

Generation of mature miRNAs takes place within two distinct cellular compartments (Figure 1): In the nucleus, miRNAs are transcribed from DNA sequences by polymerase II as pri-microRNAs, processed by the RNAse-III enzyme Drosha and its auxiliary protein DGCR8 (Pasha) into 5′ capped and poly-adenylated pre-miRNAs, which can be several kilobases long, and may comprise one (monocistronic) or several (polycistronic) miRNA precursors [23]. Pre-miRNA sequences form characteristic hairpin loop structures of ∼70 nucleotides with a two-nucleotide overhang at the 3′ end and 3′ hydroxyl and 5′ phosphate groups [23–25]. Alternatively, short introns forming a hairpin-like structure called mirtrons can be spliced in a Drosha-independent manner to be further processed into a pre-miRNA [26]. These recently discovered features illustrate unexpected flexibility and highlight how alternative RNA processing can encode multiple functions by individual transcripts. Pre-miRNAs are shuttled from the nucleus into the cytosol by Exportin-5 transporter molecules where the RNase-III enzyme *Dicer* cleaves them towards biologically active mature duplex single-stranded miRNAs (∼18–25 nucleotides) [23]. Both single-stranded miRNAs derived from the 5´ arm (-5p) and 3´ arm (-3p) of the precursor can become integrated with Argonaute (Ago) proteins to form the RNA-Induced Silencing Complex (RISC, see Figure 1; [27]). Depending on the degree of homology, the miRNA induces translational repression (incomplete match) or target mRNA degradation (full match, not in mammals). More than 50% of mammalian miRNAs are located within host genes [28]. Especially those intragenic miRNAs exhibiting a high degree of conservation between species appear to be coordinately regulated and expressed with their host genes, either with synergistic or antagonistic correlation patterns [29,30]. For example, a number of interleukin-6 (IL-6) regulated miRNAs are up-regulated in rodent models of neuropathic pain and distinct functions have been described [31]. In contrast to intragenic miRNAs, the regulatory elements for extragenic miRNA are still largely enigmatic.

### Potential prospect of miRNA patterns emerging as possible signatures for pain disorders

#### miRNA in body fluids

In addition to their intracellular location and function, miRNAs are detectable in extracellular vesicles, such as exosomes, which are released, for example, by glia cells to cargo messages to other cell types in the central nervous system. Therefore, exosomes are anticipated to represent a specific mode of intercellular communication (for review see [34,35]). In addition, they are detectable in body fluids, such as cerebrospinal fluid (CSF), blood plasma or saliva, where they can be exploited for diagnostic purposes as so called ‘liquid biopsies’. Extracellular miRNAs are emerging as important communication units not only for mental disorders but also in the pain pathway offering the advantage of long distance messaging [36,37]. Several studies propose individual miRNAs or miRNA signatures for pathological pain disorders, such as CRPS, diabetic neuropathic pain or fibromyalgia. However, the assays available for these first studies assessing miRNAs in human body fluids cover only a small number of miRNAs and the marginal overlap for the same disease when assessed at different locations dampens the great hopes in the possible value of liquid biopsies for clinical use in pain diagnostics (see Table 1).

**Table 1:** Deregulated miRNAs identified in liquid biopsies of patients with pain disorders

Disease	Detection method	Sample type	# up-regulated	# down-regulated	miRNA deregulated in minimum two studies on disease	Reference
CRPS	qPCR array	Blood	4	14	RNU48; miR-15a; miR-21; mir-25; miR-29c; miR-34a; mir-126; miR-212; mir-320B; miR-337; miR-367; miR-576; miR-645; mir-939; miR-1276; miR-1303	[38]
CRPS	qPCR array	Serum-derived exosomes	62	70		[39]
CRPS responders versus non-responders	qPCR array	Blood	1	32		[40]
CRPS responders versus non-responders	qPCR array	Serum-derived exosomes	1	8		[41]
Diabetic neuropathy	qPCR array	Serum	1	63	let-7d; let-7e; miR-28; miR-92a; miR-106a; miR-130a; miR-139; miR-150; mir-210; miR-342; miR-425; mir-486; miR-574	[42]
Diabetic neuropathy with versus without critical limb ischaemia	microarray	Plasma	7	4		[43]
Diabetic neuropathy with versus without critical limb ischaemia	microarray	Plasma	6	5		[44]
Diabetic neuropathy	qPCR array	Serum	21	0		[45]

In total, about 6000–7000 microRNA sequences have been identified, more than 2000 in humans [46]. In order to retrieve all known and possible currently unknown human miRNAs together with other ncRNAs and mRNAs, RNA sequencing (RNASeq) technologies represent the state of the art methodology for unbiased assessment of differentially expressed miRNA in patient and control cohorts [47,48]. For miRNA quantification in tissue, different experimental settings and also tissues, such as dorsal root ganglia (DRG), spinal cord or brain, require the use of appropriate reference genes and reliability is significantly higher if three different reference genes are used [49]. However, due to profound technical improvements, only unbiased RNASeq provides sufficient reliability and specificity for the discovery of disease specific miRNA patterns.

Nonetheless, for blood components, as well as nerve biopsies, a number of differentially regulated miRNAs has been identified and their target genes validated with a possible prospect to better understand disease pathophysiology (Table 2). Two up-regulated miRNAs (miR-124 and miR-155) target the histone deacetylase SIRT1, a structurally important promoter of axonal elongation, neurite outgrowth, and dendritic branching. SIRT1 also plays a role in memory formation by modulating synaptic plasticity and has protective roles in several neurodegenerative diseases [50]. The down-regulation of SIRT1 by miRNAs could thus be causally involved in neuropathic pain generation or in the exacerbation of the immune response [51]. Likewise, the increased IL-6 and VEGF expression resulting from decreased activity of miR-338-5p and miR-939, as well as miR-34a and miR-101 targeting Corticotropin releasing hormone receptor 1 (CRHR1) and Karyopherin beta 1 (KPNB1) are relevant candidates for the inflammatory component of neuropathic pain disorders [41,52,53].

**Table 2:** Deregulated miRNA in human samples including validated target genes

miRNA	Regulation	Target	Gene description	Validation	Pain disorder	Tissue	Reference
miR-124a	↑	SIRT1	Sirtuin 1	LucA	Neuropathic pain patients	CD4+ T cells	[51]
miR-132-3p	↑	GRIA1	Glutamate ionotropic receptor AMPA type subunit 1	LucA	Neuropathic pain patients	WBC / sural nerve	[54]
miR-155	↑	SIRT1	Sirtuin 1	LucA	Neuropathic pain patients	CD4+ T cells	[51]
miR-199a-3p	↑	SERPINE2	Serpin family E member 2	LucA	Diabetes type II	Plasma	[55]
miR-455-3p	↑	TUBB3	Tubulin beta 3 class III	mimic + immunofluorescence	HIV-induced polyneuropathy	Plasma	[56]
miR-34a	↓	XIST; YY1	X inactive specific transcript; YY1 transcription factor	LucA	CRPS	Blood	[57]
miR-34a	↓	CRHR1	Corticotropin releasing hormone receptor 1	LucA	CRPS	Blood	[53]
miR-101	↓	KPNB1	Karyopherin subunit beta 1	LucA	Neuropathic pain patients	Plasma / sural nerve	[58]
miR-338-5p	↓	IL6	Interleukin 6	LucA	CRPS	Plasma	[41]
miR-939	↓	VEGFA	Vascular endothelial growth factor A	LucA	CRPS	Plasma	[52]

### miRNA tissue expression in rodent models

Similar to human studies, differentially expressed miRNAs are extensively explored in preclinical neuropathic pain models [31,59–64]. In several of the routinely used models, specific miRNAs are up- or down-regulated all along the pain pathway and described in a number of recent publications and reviews [59,60,62,64–71]. The following section takes a more mechanistic approach and provides an overview of miRNAs deregulated in tissues in relevant preclinical pain models for which a relevant target gene and insight into neuropathic pain mechanisms have been validated (see Table 3).

**Table 3:** Look-up table of deregulated miRNA and target genes related to neuropathic human pain disorders or preclinical models of neuropathic pain. For ethical reasons, bilateral CCI was excluded as a model

miRNA	Regulation	Target	Gene description	Validation	Pain model	Tissue	Species	Reference
miR‐15b	↑	Bace1	Beta-secretase 1	LucA	Oxaliplatin‐induced peripheral neuropathy	DRG	Rat	[72]
miR-18a	↑	Kcna1; Kcnd3	Potassium voltage-gated channel subfamily A member 1; Potassium voltage-gated channel subfamily D member 3	LucA	SNL	DRG	Rat	[73]
miR-19a	↑	Kcna4; Kcnc4; Kcnq5; Scn1b	Potassium voltage-gated channel subfamily A member 4; Potassium voltage-gated channel subfamily C member 4; Potassium voltage-gated channel subfamily Q member 5; Sodium voltage-gated channel beta subunit 1	LucA	SNL	DRG	Rat	[73]
miR-19b	↑	Kcna4; Kcnc4; Kcnq5; Scn1b	Potassium voltage-gated channel subfamily A member 4; Potassium voltage-gated channel subfamily C member 4; Potassium voltage-gated channel subfamily Q member 5; Sodium voltage-gated channel beta subunit 1	LucA	SNL	DRG	Rat	[74]
miR-32-5p	↑	Dusp5	Dual specificity phosphatase 5	LucA	SNL	Spinal cord / microglia	Rat	[75]
miR-92a	↑	Kcnc4; Dpp10	Potassium voltage-gated channel subfamily C member 4; Dipeptidyl peptidase like 10	LucA	SNL	DRG	Rat	[73]
miR-124a	↑	SIRT1	Sirtuin 1	LucA	Neuropathic pain patients	CD4+ T cells	Human	[51]
miR-132-3p	↑	GRIA1	Glutamate ionotropic receptor AMPA type subunit 1	LucA	Neuropathic pain patients	WBC / sural nerve	Human	[54]
miR-132-3p	↑	Gria1	Glutamate ionotropic receptor AMPA type subunit 1	LucA	SNI	Sural nerve / spinal cord / DRG	Rat	[54]
miR-146a-5p	↑	Traf6	TNF receptor-associated factor 6	LucA	SNL	Spinal astrocytes	Mouse	[76]
miR-155	↑	Socs1	Suppressor of cytokine signalling 1	LucA	CCI	Spinal cord / microglia	Rat	[77]
miR-155	↑	SIRT1	Sirtuin 1	LucA	Neuropathic pain patients	CD4+ T cells	Human	[51]
miR-183-5p	↑	Cldn1	Claudin 1	mimic + WB	Perineural injection of sciatic nerve with recombinant tissue plasminogen activator	Sciatic nerve	Rat	[78]
miR-195	↑	Ptch1	Patched 1	LucA	Infraorbital nerve CCI	Brain stem	Rat	[79]
miR-195	↑	Atg14	Autophagy related 14	LucA	SNL	Spinal cord / microglia	Rat	[80]
miR-199a-3p	↑	SERPINE2	Serpin family E member 2	LucA	Diabetes type II	Plasma	Human	[55]
miR-218	↑	Socs3	Suppressor of cytokine signalling 3	LucA	CCI	Spinal cord / microglia	Rat	[81]
miR-221	↑	Socs1	Suppressor of cytokine signalling 1	LucA	CCI	Spinal cord / microglia	Rat	[82]
miR-449a	↑	Pparg	Peroxisome proliferator-activated receptor gamma	LucA	SCI	Spinal cord	Rat	[83]
miR-455-3p	↑	TUBB3	Tubulin beta 3 class III	mimic + immunofluorescence	HIV-induced polyneuropathy	Plasma	Human	[56]
miR-500	↑	Gad1	Glutamate decarboxylase 1	LucA	Paclitaxel-induced neuropathic pain	Spinal dorsal horn	Rat	[84]
miR-7a	↓	Scn2b	Sodium voltage-gated channel beta subunit 2	LucA	SNL	DRG	Rat	[85]
miR-7a	↓	Nefl	Neurofilament light	LucA	SNL	DRG	Rat	[86]
miR-9	↓	Foxp1	Forkhead box P1	mimic + WB	Sciatic nerve crush	DRG	Mouse	[87]
miR-19a	↓	Mecp2	Methyl CpG binding protein 2	LucA	SNI	DRG	Mouse	[88]
miR-20b-5p	↓	Akt3	AKT serine/threonine kinase 3	LucA	CCI	Spinal cord	Rat	[89]
miR-21-5p	↓	Timp3; Ccl1	TIMP metallopeptidase inhibitor 3; C-C motif chemokine ligand 1	LucA	CCI	Spinal cord	Rat	[90]
miR-23a-3p	↓	Cxcr4	Chemokine (C-X-C motif) receptor 4	LucA	SNL	Spinal cord	Mouse	[91]
miR-23b	↓	Nox4	NADPH oxidase 4	LucA	Traumatic SCI (neuropathic pain)	Spinal cord	Mouse	[92]
miR-26a-5p	↓	Mapk6	Mitogen-activated protein kinase 6	LucA	CCI	Spinal cord	Rat	[93]
miR-30b	↓	Scn9a	Sodium voltage-gated channel alpha subunit 9	LucA	SNI	DRG	rat	[94]
miR-30b	↓	Scn3a	Sodium voltage-gated channel alpha subunit 3	LucA	SNL	DRG	Rat	[95]
miR-34a	↓	Scn2b; Vamp2	Sodium voltage-gated channel beta subunit 2; Vesicle-associated membrane protein 2	LucA	CCI	DRG	Rat	[96]
miR-34a	↓	XIST; YY1	X inactive specific transcript; YY1 transcription factor	LucA	CRPS	Blood	Human	[57]
miR-34a	↓	CRHR1	Corticotropin releasing hormone receptor 1	LucA	CRPS	Blood	Human	[53]
miR-34c	↓	Nlrp3	NLR family, pyrin domain containing 3	LucA	CCI	Spinal cord	Mouse	[97]
miR-96	↓	Scn3a	Sodium voltage-gated channel alpha subunit 3	mimic + WB	CCI	DRG	Rat	[98]
miR-101	↓	KPNB1	Karyopherin subunit beta 1	LucA	Neuropathic pain patients	Plasma / sural nerve	Human	[58]
miR-132	↓	Mecp2	Methyl CpG binding protein 2	LucA	SNI	DRG	Mouse	[88]
miR-141	↓	Hmgb1	High mobility group box 1	LucA	CCI	DRG	Rat	[99]
miR–142–3p	↓	Hmgb1	High mobility group box 1	LucA	SNL	DRG	Mouse	[100]
miR-145	↓	Akt3	AKT serine/threonine kinase 3	LucA	CCI	DRG	Rat	[101]
miR-145	↓	Rreb1	Ras responsive element binding protein 1	LucA	CCI	Spinal cord	Rat	[102]
miR-182-5p	↓	Ephb1	Eph receptor B1	LucA	CCI	Spinal cord	Mouse	[103]
miR-183	↓	Mtor	Mechanistic target of rapamycin kinase	LucA	CCI	Spinal cord	Rat	[104]
miR-183	↓	Scn3a; Bdnf	Sodium voltage-gated channel alpha subunit 3; Brain-derived neurotrophic factor	mimic + qPCR	SNL	DRG	Rat	[105]
miR-183-5p	↓	Kcnk2	Potassium two pore domain channel subfamily K member 2 (Trek1)	LucA	CCI	DRG	Rat	[106]
miR-186-5p	↓	Cxcl13	Chemokine (C-X-C motif) ligand 13	LucA	SNL	Spinal cord	Mouse	[107]
miR-190a-5p	↓	Slc17a6	Solute carrier family 17 (sodium-dependent inorganic phosphate cotransporter), member 6 (Vglut2)	LucA	Diabethic neuropathy	Spinal cord	Mouse	[108]
miR-200b	↓	Zeb1	Zinc finger E-box binding homeobox 1	LucA	CCI	Spinal cord / microglia	Rat	[109]
miR-206	↓	Bdnf	Brain-derived neurotrophic factor	LucA	CCI	DRG	Rat	[110]
miR-206-3p	↓	Hdac4	Histone deacetylase 4	LucA	CCI	DRG	Rat	[111]
miR-301	↓	Mecp2	Methyl CpG binding protein 2	LucA	SNI	DRG	Mouse	[88]
miR-338-5p	↓	IL6	Interleukin 6	LucA	CRPS	Plasma	Human	[41]
miR-362-3p	↓	Pax2	Paired box 2	LucA	SCI	Spinal cord	Rat	[112]
miR-429	↓	Zeb1	Zinc finger E-box binding homeobox 1	LucA	CCI	Spinal cord/microglia	Rat	[109]
miR–449a	↓	Trpa1; Kcnma1	Transient receptor potential cation channel, subfamily A, member 1; Potassium large conductance calcium-activated channel, subfamily M, alpha member 1	mimic + qPCR	SNI	DRG	Mouse	[113]
miR-539	↓	Grin2b	Glutamate ionotropic receptor NMDA type subunit 2B	mimic + WB	CCI	ACC	Rat	[114]
miR-939	↓	VEGFA	Vascular endothelial growth factor A	LucA	CRPS	Plasma	Human	[52]

Abbreviations: CCI, chronic constriction injury; CRPS, chronic regional pain syndrome; SCI, spinal cord injury; SNI, spared nerve injury; SNL, sciatic nerve ligation.

### Mechanisms of miRNA action

Intracellular miRNAs suppress gene expression through tightly regulated steps within the microprocessor complex. For this, the miRNA within the ‘activated’ RNA-induced silencing complex (RISC) attaches to the 3′-untranslated region (UTR) of a given target mRNA primarily through its heptameric 5′ seed region (positions 2–8). One given mRNA harbouring one or several miRNA-binding sites in its 3′UTR can be regulated by various miRNAs. miRNA binding to the target gene’s miRNA recognition element (MRE) with full complementarity (which is rare in mammals) leads to destabilization and degradation (see also Figure 1); if the complementarity is incomplete, binding induces translational repression [115]. Protein expression may be down-regulated, although mRNA levels may remain unaltered in case of incomplete complementarity. miRNAs regularly target a multitude of target genes at the same time and thus may regulate entire signalling networks within one cell. In addition to their regulated target mRNAs, miRNAs hybridize with pseudogenes or circular RNAs (circRNAs) acting as endogenous miRNA neutralizing sponges, which inhibit or limit intracellular miRNA effects [116]. An unconventional role of extracellular miRNAs for rapid excitation of nociceptor neurons has been discovered recently: miRNA-let-7b induces rapid inward currents and excitation of nociceptors. These responses require the GUUGUGU motif, only occur in neurons co-expressing TLR7 and TRPA1, and are abolished in mice lacking Tlr7 or Trpa1. Thus, extracellular miRNAs may in addition to their regulatory function act as aptamers with a role as pain mediators via activating TLR7/TRPA1 in nociceptor neurons [117].

Since the first publication on analgesic miR-124 effects, miRNA regulation has attracted increasing attention in the pain field. However, the increasing number of reports raises the problem how expression patterns and mechanisms can be interpreted with a more global perspective. In order to warrant the highest degree of stringency, only miRNAs are addressed in this review based on publications validating miRNA expression together with direct regulation of target genes related to pain processing (Table 3).

#### miRNAs deregulated in the peripheral nerve

**Figure 2:**
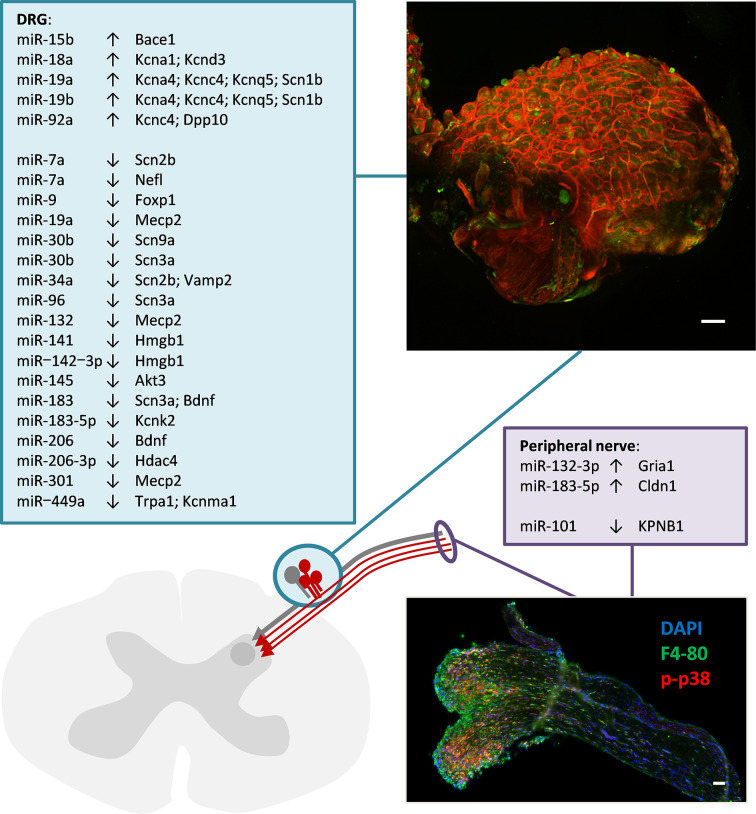
Up- or down-regulated miRNAs in peripheral nerve or sensory ganglia and their target genes that are associated with neuropathic pain *Akt3*: AKT serine/threonine kinase 3; *Bace1*: Beta-secretase 1; *Bdnf*: Brain-derived neurotrophic factor; *Dpp10*: Dipeptidyl peptidase like 10; *Foxp1*: Forkhead box P1; *Hdac4*: Histone deacetylase 4; *Hmgb1*: High mobility group box 1; *Kcna1*: Potassium voltage-gated channel subfamily A member 1; *Kcna4*: Potassium voltage-gated channel subfamily A member 4; *Kcnd3*: Potassium voltage-gated channel subfamily D member 3; *Kcnk2*: Potassium two pore domain channel subfamily K member 2 (Trek1); *Kcnma1*: Potassium large conductance calcium-activated channel, subfamily M, alpha member 1; *Kcnc4*: Potassium voltage-gated channel subfamily C member 4; *Kcnq5*: Potassium voltage-gated channel subfamily Q member 5; *Mecp2*: Methyl CpG binding protein 2; *Nefl*: Neurofilament light; *Scn1b*: Sodium voltage-gated channel beta subunit 1; *Scn2b*: Sodium voltage-gated channel beta subunit 2; *Scn3a*: Sodium voltage-gated channel alpha subunit 3; *Scn9a*: Sodium voltage-gated channel alpha subunit 9; *Trpa1*: Transient receptor potential cation channel, subfamily A, member 1; *Vamp2*: Vesicle-associated membrane protein 2. Scale bar: 100 µm (micrographs were kindly provided by M. Langeslag)

Only few studies address miRNAs in peripheral nerve tissue where axons, Schwann cells, connective tissue, cells of the vasculature and resident as well as transient immune cells can be possible sources (Figure 2). Up-regulation of miR-132-3p may target the ionotropic glutamate receptor AMPA type subunit 1 (Gria1) [54]. This has been identified as a microglial gene potentially linked to the maintenance of neuropathic pain [118]. Another up-regulated miRNA, miR-183-5p, targets Claudin 1, a tight junction protein that maintains the blood brain/blood nerve barrier and is down-regulated after nerve injury [78,119]. Along these lines, down-regulation of miR-101 by targeting Importin beta 1 (also known as Karyopherin beta 1, KPNB1) may release a brake on importin expression and augment the accessibility of axons to exosomal cargo as a mechanisms to foster nerve regeneration but possibly also for proalgesic factors [58,120].

#### miRNAs deregulated in the dorsal root and trigeminal ganglia

miRNA expression in the dorsal root or trigeminal ganglia (Figure 2 andTable 3) can be deregulated in various cell types, such as neurons, Schwann cells, resident or invading immune cells or even the vasculature. The most intensely studied sources are peptidergic and non-peptidergic primary afferent nociceptors. Conditional deletion of the miRNA-maturation enzyme Dicer exclusively in neurons expressing the nociceptor specific sodium channel Nav1.8 critically affects neuronal excitability [121] and increasing evidence suggests that several miRNAs directly or indirectly modulate neuron function (Table 3). Particular miRNAs are deregulated in peripheral neurons after nerve injury giving rise to deregulation of miRNA targeted ion channel and metabotropic receptor transcripts that presumably causes nociceptor dysfunction [117,121–123].

The most intensely investigated miRNA in the DRG is presently miR-21 that is expressed in neurons and up-regulated in several neuropathic pain models. Both intrathecal delivery of a miR-21-5p antagomir and conditional deletion of miR-21 in sensory neurons reduce neuropathic hypersensitivity [36]. As a new mechanism of action miR-21 cargo from neurons to immune cells via exosomes has been introduced recently: following capsaicin activation, miR-21-5p containing exosomes are released from cultured DRG and phagocytosed by macrophages in which the resulting increase in miR-21-5p levels promotes a pro-inflammatory phenotype. Both up-regulation and release of miR-21 contribute to sensory neuron-macrophage communication after damage to the peripheral nerve [36]. Since intrathecal miR-21 injection induces pain hypersensitivity in wild-type mice but not in mice with a global deletion of toll-like receptor 8 (Tlr8^−/−^), the TLR8 receptor appears to act as a downstream effector of miR-21 to maintain neuropathic pain; however, a direct targeting of the Tlr8 gene has not been validated yet [124].

miR-18, miR-19a, miR-19b as well as mir-92 are also up-regulated in neuropathic pain models and in turn down-regulate potassium channels including Kcna1, Kcna4, Kcnc4, Kcnd3 and Kcnq5 [73]. The suppression of potassium channels in general increases neuronal excitability and this may be a relevant mechanism causing nociceptor excitation and sensitization [125,126]. In line with the alterations towards hyperexcitability, several down-regulated miRNAs targeting voltage-gated sodium channels may further promote neuronal excitability by releasing the breaks on sodium channel expression: two alpha subunits of voltage-gated sodium channels, Scn3a giving rise to Na_v_1.3 and Scn9a giving rise to pain-related Na_v_1.7, are targeted by the down-regulated miRNAs miR-30b, miR-96 and miR-183, which probably contributes to up-regulation of the ion channel alpha subunits in neuropathic pain models [94,95,98,105]. In addition, down-regulated miR-7a and miR-34a targeting sodium voltage-gated channel beta subunit 2 (Scn2b) may be involved in improved trafficking of already formed alpha subunits [85,96]. Furthermore, transducer channels, such as TRPA1, can occur as a consequence of miR–449a down-regulation [113]. The only potential change counteracting these proalgesic alterations refers to the down-regulated miR-183-5p that targets potassium channel Trek1, a two pore domain potassium channel subfamily member 2 (Kcnk2), which as a leak current keeps the membrane potential hyperpolarized [106]. The up-regulation of this channel may counteract the brake set on potassium channel expression by the miR-17-92 cluster.

Alterations in enzymatic activity, inflammatory signalling pathways and epigenetic regulators are amongst the mechanisms targeted by deregulated miRNAs in DRG. For example, miR-15b up-regulation targets Beta-secretase 1 that may be involved in neuroprotective processes, whereas miR-145 down-regulation appears to release the break on Akt3 that can be targeted by miR-15a or miR-20b-5p mimics to relieve neuropathic pain [72,89,101,127]. Down-regulation of miR-183 and miR-206 contributes to the up-regulation of brain-derived neurotrophic factor (Bdnf) that is required for regenerative processes but also is an important pain modulator [105,110,128,129] (for review see [130]). Finally, several down-regulated miRNAs (miR-19a, miR-132, miR-301) appear to affect epigenetic regulatory pathways, such as Mecp2 or HDAC4 (via miR-206-3p), and dependent processes that are relevant contributors fueling pathological functions in the spinal dorsal horn but may also be relevant for dysfunction of primary afferent nociceptors [88,111,131–135].

#### miRNAs deregulated in the spinal cord

**Figure 3:**
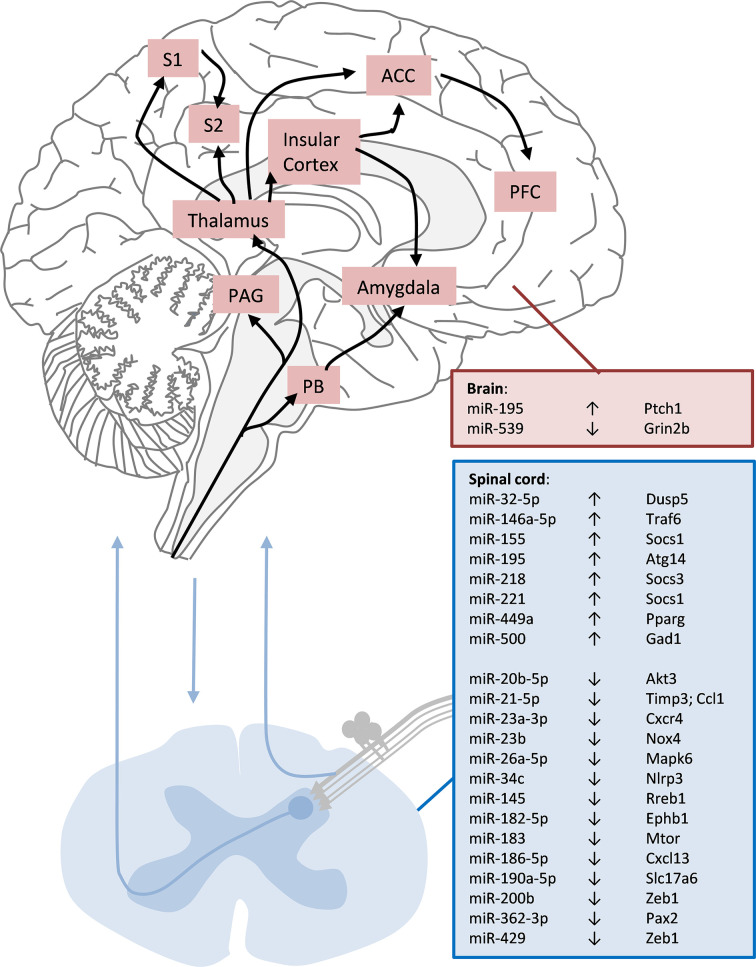
Deregulated miRNA in spinal cord and brain with the respective associated target genes Although specific brain areas are involved in different aspects of processing pain perception, miRNA expression so far has not been addressed specifically within these areas under neuropathic pain conditions. *Akt3*: AKT serine/threonine kinase 3; *Atg14*: Autophagy related 14; *Dusp5*: Dual specificity phosphatase; *Ccl1*: C-C motif chemokine ligand 1; *Cxcl13*: Chemokine (C-X-C motif) ligand 13; *Cxcr4*: Chemokine (C-X-C motif) receptor 4; *Ephb1*: Eph receptor B1; *Gad1*: Glutamate decarboxylase 1; *Grin2b*: Glutamate ionotropic receptor NMDA type subunit 2B; *Mapk6*: Mitogen-activated protein kinase 6; *Mtor*: Mechanistic target of rapamycin kinase; *Nlrp3*: NLR family, pyrin domain containing 3; *Nox4*: NADPH oxidase 4; *Pax2*: Paired box 2; *Pparg*: Peroxisome proliferator-activated receptor gamma; *Ptch1*: Patched 1; *Rreb1*: Ras responsive element binding protein 1; *Slc17a6*: Solute carrier family 17 (sodium-dependent inorganic phosphate cotransporter); *Socs1*: Suppressor of cytokine signalling 1; *Socs3*: Suppressor of cytokine signalling 3; *Timp3*: TIMP metallopeptidase inhibitor 3; *Traf6*: TNF receptor-associated factor 6; *Zeb1*: Zinc finger E-box binding homeobox 1

miR-124 was the first miRNA for which an analgesic action at spinal cord level was demonstrated and correlated with a shift in the M1/M2 microglial marker ratio towards an anti-inflammatory phenotype [136]. The functional consequences of miR-103 regulation of Ca_v_1.2 calcium channels and intrinsic excitability of spinal projection neurons have also been demonstrated [123]. More evidence supporting miRNA analgesic effects emerge from mice intrathecally receiving miR-124 or miR-103, which are reported to prevent and treat persistent inflammatory and neuropathic pain [123,136]. Despite the fact that these miRNA treatments reduce signatures of synaptic modification, neuroinflammation and microglial response, the full extent and the mechanisms of the analgesic effect are not fully understood to date [34,123,136,137]. However, several miRNAs have been found deregulated in rodent pain models together, mainly in microglia, with validated downstream targets with a potential relevance to pain signalling (Figure 3).

Deregulated miRNAs in the spinal cord (Figure 3) can emerge from various sources, such as neurons, microglia, astroglia or even the vasculature. The most intensely investigated sources are microglia that react to maintained nociceptive input to the spinal cord with proliferation and a change in phenotype and activity (microgliosis). In microglia, a number of up-regulated miRNA (miR-155, miR-218 and miR-221) can inhibit the expression of suppressors of cytokine signalling, such as Socs1, and may promote inflammatory signatures, such as microgliosis [77,81,82]. A novel regulator of microglia, Dual specificity phosphatase 5 (Dusp5) is regulated by miR-32-5p, and this is involved in the regulation of neuroinflammatory processes, such as cytokine release in the spinal cord [75].

At the same time several miRNAs, such as miR-200b and miR-429, are down-regulated in microglia after nerve injury, and Zeb1 (Zinc finger E-box binding homeobox 1) has been identified as a target of these two miRNAs [109]. Zeb family members are essential for the developing nociceptors in the DRG [138]. ZEB1 overexpression regulates the microglia response after ischemic stroke and in turn inhibits the production of astrocytic CXCL1 which leads to a decline in neutrophil infiltration, thereby reducing CNS inflammation. This suggests involvement of miR-200b and miR-429 suppression in the resolution of neurological injury [139]. In contrast, miR-21-5p targeting the chemokine Ccl1 [90], miR-23a-3p targeting Cxcr4 [91], miR-34 targeting Nlrp3 (NLR family, pyrin domain containing 3) [97], and miR-186-5p targeting Cxcl13 [107] are down-regulated leading to increased expression of inflammatory mediators and an augmentation of inflammatory processes in the spinal cord. Other down-regulated miRNAs (miR-145, miR-183) at the same time may release the suppression of key components affecting cell morphology and microglia function via transcription factor Rreb1 [102,140] or mTOR [104,141] together with miR-195 up-regulation, which suppresses Autophagy related 14 (Atg14 [80]). These microRNAs appear to orchestrate spinal neuroinflammation related to neuropathic pain. Another negative-feedback regulator of the astrocyte-mediated inflammatory response to injury is miR-146a, which is up-regulated in astrocytes following nerve injury, and targets TNF receptor-associated factor 6 (Traf6) [76,142]. Persistent up-regulation of Traf6 in spinal cord astrocytes in the late phase after nerve injury maintains neuropathic pain by integrating TNF-α and IL-1β signaling and activating the JNK/CCL2 pathway and increased miR-146a expression can set a brake to the neuroinflammatory component maintained by Traf6 [76,143].

**Figure 4:**
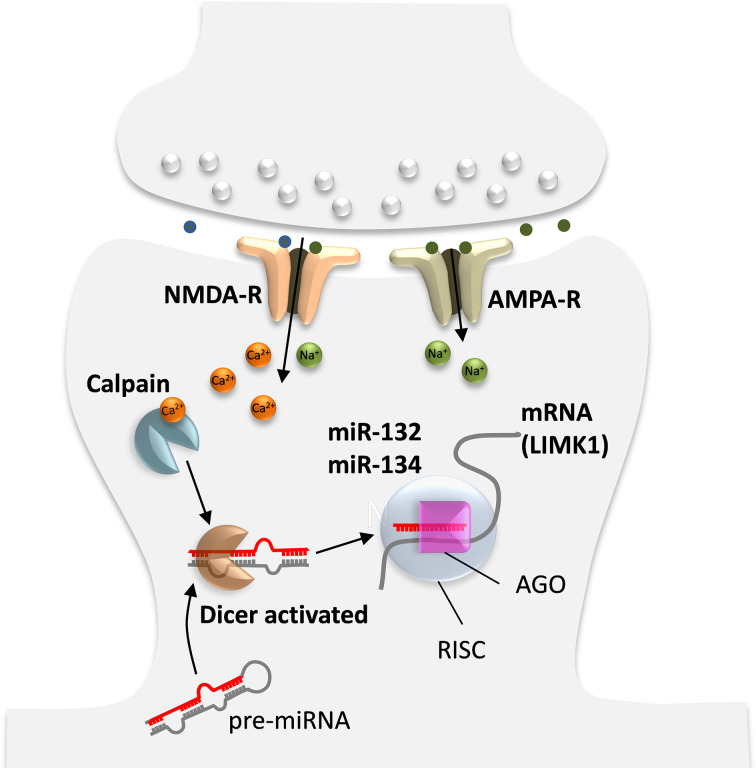
Activity dependent generation of miRNA in neurons affecting synapse specific protein synthesis Adapted from [145].

In addition, miRNAs are generated and act within neurons, and there are several possible mechanisms for activity-dependent miRNA regulation. First, upon strong synaptic input, Ca^2+^ influx through NMDA receptors activates the Ca^2+^-dependent enzyme calpain that can liberate Dicer from postsynaptic densities and stimulate Dicer RNAse III activity to facilitate processing of pre-miRNAs into mature miRNAs (Figure 4) [144]. Second, increased intracellular Ca^2+^ can induce *de novo* miRNA transcription. Third, although less is known about the mechanism of miRNA degradation, another possible mechanism controlling mature miRNA expression may be the activity-dependent degradation of the RISC component MOV10 by the proteasome. However, it is still not sufficiently clear whether MOV10 degradation promotes the disassembly of RISC and thus augments the turnover of miRNAs (for review see [145]).

Of particular interest and importance in the spinal dorsal horn (SDH) are up-regulated miRNAs targeting inhibitory ion channels, such as GABA or K^+^ channels. In the chronic constriction injury (CCI) model, miR-182-5p is down-regulated and targets Ephb1 [103]. This is of particular interest, since ephrinB–EphB receptor signalling plays a critical role in induction and maintenance of neuropathic pain by regulating neural excitability and synaptic plasticity in the DRG and the SDH [146]. Up-regulation of miR-449a via targeting peroxisome proliferator-activated receptor gamma (Pparg) and reduced Pparg expression can aggravate the increased neuroexcitability and neuroexcitotoxicity associated with neuropathic pain [83,147]. Increased excitatory activity in SDH may be further fueled by the release of glutamate transporter vGluT2 (Slc17a6, Solute carrier family 17 member 6) expression and augmentation by down-regulated miR-190a-5p [108,148–150]. At the same time, glutamate decarboxylase 1 (Gad1) regulates GABA synthesis and transport [151], and reduced expression of Gad1 impairs the function of GABAergic synapses, which appears to be induced by up-regulation of miR-500 [84]. The transcription factor Paired box 2 (Pax2) is necessary for the GABAergic differentiation [151] and a loss of SDH GABAergic interneurons causes reduced GABAergic tone that contributes to neuropathic pain [152]. Therefore, the down-regulation of miR-362-3p targeting Pax2 may rather be a compensatory change to restore GABAergic signaling in the SDH [112]. These processes are further enhanced by the down-regulation of three miRNAs (miR-20b-5p, miR-23 and miR-26a-5p) leading to the increased expression of the kinases Akt3, Nox4 and Mapk6 whose relevance for neuropathic pain is well accepted [89,92,93]. Together, miRNAs are emerging as major controllers of neuro-immune processes in the SDH by switching neurons as well as non-neuronal cells into proalgesic modes of action and promoting the development and maintenance of signatures aggravating neuropathic pain at spinal cord level.

#### miRNAs deregulated in the brain

miRNAs act at the neuro-immune interface which controls neuronal plasticity and memory but also are linked to the etiology of anxiety and mood disorders [12,13,34]. Deficits in the interaction of immune cells and neurons together with cognitive and emotional alterations in patients with neuropathic or neurogenic pain syndromes are hypothesized to converge on ncRNA deregulated mechanisms along the entire neuraxis, and alterations in ncRNAs expression may account for the variation of susceptibility to certain types of pain or even for the responsiveness to analgesics and development of opioid tolerance [153].

As in the spinal cord, neuronal and non-neuronal cells contribute to potential disease specific miRNA patterns in brain regions that are relevant for the processing of painful stimuli. Brain specific miRNAs are emerging as regulators of cognition, neuronal plasticity and memory, affecting synapse structure and function, and specific miRNAs not only control cognition and emotional processes but also neuro-immune communication in the brain [13,154]. In general, happiness, anxiety and depression seem to depend on miRNA expression levels, and specific miRNAs are deregulated in depression, anxiety, and preclinical models of psychological stress. Moreover psychoactive agents including antidepressants and mood stabilizers utilize miRNAs as downstream effectors [12]. In the brain, AMPA-mediated synaptic transmission is reduced by neuronal over-expression of miR-125b and increased by miR-132 due to differential regulation of glutamate NR2A and NR2B receptor mRNA [155]. Other glutamate receptor subunits in the brain are regulated by dopamine through miR-181a, which has been associated with the pain system [156]. In particular, miR-132 is a highly interesting brain specific miRNA, since it is up-regulated by BDNF and other growth factors in cortical neurons resulting in an increased expression of synaptic glutamate receptors NR2A, NR2B and AMPA GluR1 [157,158]. In the hippocampus, miR-132 targets acetylcholinesterase and this is relevant for stress-induced cognitive deficits. In the amygdala miR-34 is associated with the repression of stress-induced anxiety [159,160]. In neuropathic pain, maladaptive responses of the nucleus accumbens have been associated with deregulated miRNAs [66]. Endogenous pain control systems including GABAergic and opioidergic synaptic signals are down-regulated by miRNAs, such as miR-134 or miR-181a, with some of them linking miRNAs like let-7 or miR-339 to opioid tolerance [161–164]. Despite the increasing number of reports, only few studies validate the deregulated miRNAs acting on specific target genes in the brain in neuropathic pain models (Figure 3). Down-regulation of miR-539 can release the brake on the expression of Grin2b (Glutamate ionotropic receptor NMDA type subunit 2B) and enhance the formation of pain memory in the anterior cingulate cortex [114] and also in the brain stem, up-regulation of miR-195 and its target gene patched 1 (Ptch1) has been reported [79]. Altogether, although relevant roles for miRNA regulated processes in the pathogenesis of neuropathic pain can be anticipated also for relevant brain areas this field of pain research is still in its infancy.

## Long ncRNAs

### Synthesis and function

Long ncRNAs (lncRNAs) of more than 200 nucleotides are the second most studied group of ncRNAs. Currently 172,216 human and 131,697 mouse transcripts are annotated in the systematic database NONCODEv5 [165], whereas the curated knowledgebase LncBook documents 270,044 human lncRNAs [166]. Depending on their genomic location, lncRNA sequences can be intronic, natural antisense transcripts (NATs), sense, extragenic, enhancer, promoter and bidirectional, and can have a linear or circular structure (circRNAs [167,168]). lncRNA expression patterns highly depend on cell and tissue type as well as on developmental or disease states [169]. In general, lncRNA genes resemble protein coding genes, as their transcription follows similar rules [169,170]. lncRNAs can be capped at their 5′ end, polyadenylated at their 3′ end, alternatively adenylated or not, and can undergo alternative splicing [167,171]. Besides similar transcriptional mechanisms to protein coding genes, lncRNA biogenesis processes can include cleavage by ribonuclease P to form triple helical structures and the formation of circular structures [168]. Their localization in the nucleus, cytoplasm, mitochondria, ribosomes, extracellular membranes and exosomes defines and controls their functions in cellular processes, such as transcriptional, post-transcriptional and epigenetic regulation [169,170,172]. lncRNAs can act as decoys, competing endogenous RNAs (ceRNAs), guides, scaffolds and signals [167,168,173]. In the nucleus, lncRNAs can affect transcription via epigenetic mechanisms and chromatin remodeling as well as by stabilizing mRNA and changing mRNA splicing [173]. As decoys, lncRNAs can bind to proteins, such as transcription factors or RNA-binding proteins, making them unavailable to perform their actions, which can result in transcriptional inhibition and mRNA degradation [167]. Furthermore, lncRNAs can act as competing endogenous RNA (ceRNA) sponging miRNAs and making them unavailable for interaction with their target genes. In contrast to miRNAs, lncRNAs interact with single targets to up- or down-regulate expression levels. Due to the various mechanisms of actions and the high number of lncRNAs, their precise functions and interconnections are not fully unveiled. Their implications in pain conditions have been addressed mostly in animal models, providing a firm indication on the importance of lncRNAs and circRNAs in the establishment and development of pain; however, the precise mechanisms are not fully elucidated.

### lncRNAs as possible signatures for pain disorders

lncRNAs are deregulated in diabetic peripheral neuropathy and CRPS [57,137,174–176], and similar to miRNAs, they can be detected in liquid biopsies (Table 4). The most studied lncRNA in DNP is NONRATT021972 which is up-regulated in the serum and correlates with higher neuropathic pain scores, indicating its potential as a biomarker [137,177]. Another up-regulated ncRNA in Type 2 diabetes mellitus and DNP serum is uc.48+ that is involved in purinergic receptor-mediated responses [74,178]. Amongst 1327 deregulated lncRNAs in the PBMCs of female patients with DNP, MALAT1, H19, PVT1 and MIR143HG could potentially qualify as biomarkers [174]. In female CRPS patients, XIST appears as a promising indicator of poor responsiveness to ketamine [57]. XIST acts as a ceRNA on miR-34a, thus enhancing expression levels of the transcription factor YY1 promoting XIST expression in an autoregulatory loop [57]. CircRNAs are much less studied in patients with neuropathic pain and currently there is only one study showing that circHIPK3 expression positively correlates with the severity of neuropathic pain in diabetic patients [176].

**Table 4:** Deregulated lncRNAs identified in liquid biopsies of patients with pain disorders

Disease	Detection method	Sample type	Up-regulation	Down-regulation	Deregulated lncRNA or circRNA	Reference
Diabetic neuropathy	microarray	PBMCs	256	1071	MALAT1, H19, PVT1, MIR143HG	[174]
Diabetic neuropathy	RT-qPCR	Serum	1	na	NONRATT021972	[137]
Diabetic neuropathy	RT-qPCR	Serum	1	na	NONRATT021972	[177]
Diabetic neuropathy	RT-qPCR	Serum	1	na	uc.48+	[179]
Diabetic neuropathy	RT-qPCR	Serum	1	na	uc.48+	[178]
Diabetic neuropathy	RT-qPCR	Serum	1	na	circHIPK3	[176]
CRPS in female patients	RT-qPCR	Whole blood	1	na	XIST	[57]

### lncRNA tissue expression in animal models

lncRNAs and circRNAs are differentially expressed in animal pain models in sciatic nerve, DRG and spinal cord [180–185]. Most of these studies combine lncRNA and mRNA expression analyses including complex bioinformatics to generate networks of potential functional interactions [180,186,187]; however, only few have been explored using a more mechanistic approach (Table 5). Differential expression of lncRNAs upon peripheral nerve injuries is time-dependent and varies between strains and species [182,183,185]. LncRNAs play important roles in neuropathic pain processes not only in neurons, but also in non-neuronal cells, such as Schwann cells, satellite glial cells, macrophages and microglia [74,83,185,188–191].

**Table 5:** Deregulated lncRNA and target genes related to neuropathic human pain disorders or preclinical models of neuropathic pain. For ethical reasons, bilateral CCI was excluded as a model

lncRNA	Regulation	Target	Gene description	Validation	Pain model	Tissue	Species	Mechanism	Reference
**Competing endogenous RNA**									
TUSC7	↓	miR-449a	microRNA 449a	RIP	SCI	Spinal cord	Rat	lncRNA–miRNA interaction	[83]
XIST	↑	miR-34a	microRNA 34a	LucA	CRPS; CFA-induced inflammation	Whole blood	Human; mouse	lncRNA–miRNA interaction	[57]
circHIPK3	↑	miR-124	microRNA 124	LucA/RNA pull down	DNP	Serum; DRG	Human;rat	lncRNA–miRNA interaction	[176]
**Upregulation of target gene**									
BC168687	↑	Trpv1	Transient receptor potential vanilloid type 1	siRNA/WB, immunostaining	DPN	DRG	Rat	Not specified	[192]
BC168687	↑	P2rx7	Purinergic receptor P2X 7	siRNA/RT-qPCR, WB, immunostaining	DNP	Satellite glial cells cultures	Rat	Not specified	[191]
BC168687	↑	P2rx7	Purinergic receptor P2X 7	siRNA/WB, immunostaining	DPN	DRG	Rat	Not specified	[193]
MRAK009713	↑	P2rx3	Purinergic receptor P2X 3	siRNA/WB, RIP, immunostaining	CCI	DRG	Rat	lncRNA–protein interaction	[194]
NONRATT021972	↑	P2rx3	Purinergic receptor P2X 3	siRNA/WB, immunostaining	DPN	DRG	Rat	Not specified	[177]
NONRATT021972	↑	P2rx7	Purinergic receptor P2X 7	siRNA/WB, immunostaining	DPN	DRG	Rat	lncRNA–mRNA prediction	[195]
PKIA-AS1	↑	CDK6	Cyclin dependent kinase 6	LucA; siRNA/WB; RIP	SNL	Spinal cord	Rat	Enhancement of promotor activity	[196]
uc.48+	↑	P2rx3	Purinergic receptor P2X 3	siRNA/WB, immunostaining	DPN	DRG	Rat	Not specified	[178]
uc.48+	↑	P2rx7	Purinergic receptor P2X 7	siRNA/WB, immunostaining; RIP	CCI-ION	Trigeminal ganglia	Rat	lncRNA–protein interaction	[197]
**Down-regulation of target gene**									
Kcna2-NAT	↑	Kcna2	Potassium voltage-gated channel subfamily A member 2	LucA; overexpression, WB	SNL, CCI, sciatic nerve axotomy	DRG	Rat	lncRNA–mRNA interaction	[198]
Egr2-NAT	↑	Egr2	Early growth response 2	Overexpression; anti-GapMers; ChIP/WB; RIP/WB	Sciatic nerve injury	DRG: sciatic nerve	Mouse	Chromatin remodelling complex recruitment	[199]

Abbreviations: CCI, chronic constriction injury; CCI-ION, chronic constriction injury of the infraorbital nerve; CFA, Complete Freund’s adjuvant; CRPS, chronic regional pain syndrome; DPN, diabetic peripheral neuropathy; DRG, dorsal root ganglia; LucA, luciferase assay; RIP, RNA immunoprecipitation; SCI, spinal cord injury; SNL, sciatic nerve ligation; WB, Western blot.

#### lncRNAs deregulated in the peripheral nerve

Differential expression of lncRNAs and circRNAs in peripheral nerves in neuropathic pain models have not been addressed in detail. Deregulated lncRNAs are reported in the sciatic nerve after a sciatic nerve crush [185]. This model is used to explore nerve regeneration and numerous differentially expressed lncRNAs were associated with inflammatory and immune responses, which could possibly be relevant for neuropathic pain [185]. Particularly, NONMMUG014387 enhances the proliferation of cultured mouse Schwann cells, presumably via cis-upregulation of collagene triple helix repeat containing 1 (Cthrc1) [185], whereas the antisense to the Egr2 promoter ncRNA (Egr2-antisense-RNA) down-regulates Egr2 and promotes demyelination upon peripheral nerve injury by an epigenetic mechanism, in which a chromatin remodeling complex is assembled at the Egr2 promoter [199].

#### lncRNAs deregulated in the dorsal root and trigeminal ganglia

Differentially expressed lncRNAs and their functions are intensely studied in rodent neuropathic pain models, whereas circRNAs have not been addressed yet [200,201]. lncRNAs down-regulate voltage-gated channels and one of the first lncRNAs found to be implicated in pain is the endogenous voltage-gated potassium channel (K_v_) Kcna2-antisense-RNA (NAT), which upon nerve injury is transcriptionally induced by myeloid zinc finger protein 1 (MZF1) and specifically targets and down-regulates Kcna2, resulting in reduced potassium currents and increased DRG excitability [202]. Other up-regulated lncRNAs, such as BC168687 [192,193], MRAK009713 [194], NONRATT021972 [177,178,195] and uc.48+ [178,179], contribute to mechanical hypersensitivity via cell-type specific interactions and induction of different purinergic receptors known for their pain modulating properties. For example, NONRATT021972 and uc.48+ up-regulate the ionotropic purinoreceptor P2X_3_ in small-medium DRG neurons but P2X_7_ in satellite glia cells [177–179,195]. BC168687 up-regulates TRPV1 and MRAK009713 promotes the up-regulation of P2X_3_ receptor in DRG neurons [192,194]. Additionally, uc.48+ inhibition alleviates mechanical hypersensitivity in a rat model for trigeminal neuralgia, by inhibiting the expression of P2X_7_ receptor in glial cells within the trigeminal ganglia [203]. In addition, uc.48+ appears to exert inhibitory effects on neuroregeneration since inhibition of uc.127 enhances the outgrowth of DRG neurons *in vitro* [204].

#### lncRNAs deregulated in the spinal cord and brain

Most of the studies focus on whole spinal cord tissues and therefore do not distinguish between alterations occurring in neuronal and non-neuronal cells [183,184,205–208]. Nevertheless, the identification of deregulated lncRNAs and circRNAs and the computational construction of interaction-networks between lncRNAs/circRNAs–miRNAs–mRNAs provide directions for future studies and potential therapeutic targets. Interestingly, not only SNI-induced mechanical hyperalgesia but also the differential expression of lncRNAs is alleviated by the tetracycline antibiotic minocycline [205]. Few lncRNAs have been functionally investigated at the spinal cord level. PK1A-AS1 overexpression enhances pain behaviors by demethylating the promoter region and promoting the expression of CDK6, an important component in neuroinflammation and neuropathic pain, whereas PK1A-AS1 down-regulation exerts analgesic effects [196]. Of the lncRNAs that are down-regulated after peripheral nerve injury, TUSC7 may be of particular mechanistic relevance, as its up-regulation inhibits the activation of microglia by targeting miR-449a and increasing the expression of the miR-499a target gene PPAR-γ [83]. The only circRNA that has been functionally related to DPN is circHIPK3 which is up-regulated in diabetic rats, acts as a miR-124 sponge and inhibition of circHIPK3 has analgesic and anti-inflammatory effects [176]. In contrast with the emerging role of lncRNAs in the spinal cord, their importance in pain processing brain areas has so far not been mechanistically addressed.

## Synopsis

The field of ncRNAs is quickly expanding in the recent years and numerous studies have addressed and associated differentially expressed ncRNAs with neuropathic pain disorders in humans and their corresponding preclinical models. Against initial expectations, common miRNA candidates indicative of neuropathic pain in liquid biopsies have not emerged so far but lncRNAs may offer better perspectives. Whereas the suitability of miRNAs as clinically applicable biomarkers for pain disorders is still disputable, numerous studies have provided novel mechanistic insight into the role of miRNAs in the molecular sequelae involved in the pathogenesis of neuropathic pain along the entire pain pathway. Specific processes within neurons, immune cells, glia as the cellular components of the neuropathic pain triad and the communication paths between them are controlled by specific miRNAs in immune cells, neurons or glia. Therefore, nucleotide sequences mimicking or antagonizing miRNA actions can provide novel therapeutic strategies for pain treatment, provided their human homologues serve the same or at least similar functions. Similar clinical applications can be expected for tools targeting lncRNAs, which converge so far mainly on purinergic signaling pathways both in neurons and glia, and possibly even for other ncRNA species that have not been explored so far.

## Abbreviations

CRPS: complex regional pain syndrome
DPN: diabetic painful neuropathy
lncRNA: long non-coding RNA
miRNA: microRNA
ncRNA: non-coding RNA
